# Supplementary material for: ADAMS project: a genetic Association study in individuals from Diverse Ancestral backgrounds with Multiple Sclerosis based in the UK
Source: BMJ Open. 2023 May 17;13(5):e071656. doi: 10.1136/bmjopen-2023-071656 (PMC10193065; doi:10.1136/bmjopen-2023-071656)
Supplement: Supplementary data [file bmjopen-2023-071656supp001.pdf]

| Full name | Primary affiliation |
|---|---|
| Alastair J Noyce | Preventive Neurology Unit, Wolfson Institute of Population Health, Queen Mary University of London |
| Angie Dunne | Leeds Centre for Neurosciences, Leeds teaching Hospitals NHS Trust |
| Antonio Scalfari | Centre of Neuroscience, Department of Medicine, Imperial College London |
| Benjamin M Jacobs | Preventive Neurology Unit, Wolfson Institute of Population Health, Queen Mary University of London |
| Bruno Gran | Department of Neurology, Nottingham University Hospitals NHS Trust; Mental Health and Clinical Neuroscience Academic Unit, University of Nottingham School of Medicine |
| Charles A Mein | Barts and the London Genome Centre, Queen Mary University of London |
| Charlotte Sellers | Preventive Neurology Unit, Wolfson Institute of Population Health, Queen Mary University of London |
| Cord Spilker | Bradford Teaching Hospital Foundation Trust |
| David Rog | Manchester Centre for Clinical Neurosciences, Northern Care Alliance NHS Trust |
| Eli Silber | Kings College Hospital and Lewisham and Greenwich NHS Trusts |
| Elisa Visentin | Research and Innovation, Queen's Hospital, BHRUT |
| Elizabeth Lindsey Bezzina | Kings College Hospital and Lewisham and Greenwich NHS Trusts |
| Emeka Uzochukwu | Division of Psychological Medicine and Clinical Neurosciences, Cardiff University |
| Emma Tallantyre | 1. Division of Psychological Medicine and Clinical Neurosciences, Cardiff University<br>2. Department of Clinical Neurology, University Hospital of Wales, Cardiff. |
| Eva Wozniak | Barts and the London Genome Centre, Queen Mary University of London |
| Eve Sacre | Leeds Centre for Neurosciences, Leeds teaching Hospitals NHS Trust |
| Gavin Giovannoni | Preventive Neurology Unit, Wolfson Institute of Population Health, Queen Mary University of London |
| Helen L. Ford | Leeds Centre for Neurosciences, Leeds Teaching Hospitals NHS Trust |
| Huw Morris | Department of Clinical and Movement Neuroscience, UCL Queen Square Institute of Neurology, London UK |
| Jade Harris | Northern Care Alliance NHS Trust |
| Joshua Breedon | Preventive Neurology Unit, Wolfson Institute of Population Health, Queen Mary University of London |
| Judith Brooke | Northern Care Alliance NHS Trust |
| Karim L. Kreft | Department of Neurology, University Hospital of Wales, Cardiff |
| Katila George | Preventive Neurology Unit, Wolfson Institute of Population Health, Queen Mary University of London |
| Luisa Schalk | Preventive Neurology Unit, Wolfson Institute of Population Health, Queen Mary University of London |
| Martin O'Malley | Leeds Centre for Neurosciences, Leeds teaching Hospitals NHS Trust |
| Michelle Peter | NHS North Thames Genomic Laboratory Hub, Great Ormond Street Hospital for Children NHS Foundation Trust, London, UK |
| Miriam Mattoscio | Department of neuroscience, Queen's Hospital, BHRUT NHS Trust |
| Neisha Rhule | Queen Elizabeth Hospital (Lewisham and Greenwich NHS Trust) |
| Nimisha Vinod | Northern Care Alliance NHS Trust |
| Outi Quinn | Bradford Teaching Hospital Foundation Trust |
| Ramya Shamji | Research and Innovation, Queen's Hospital, BHRUT |
| Rashmi Kaimal | Preventive Neurology Unit, Wolfson Institute of Population Health, Queen Mary University of London |
| Rod Middleton | Population Data Science, Swansea University Medical School, Swansea |
| Roxanne Murray | Preventive Neurology Unit, Wolfson Institute of Population Health, Queen Mary University of London |
| Ruth Bellfield | Bradford Teaching Hospital Foundation Trust |
| Ruth Dobson | Preventive Neurology Unit, Wolfson Institute of Population Health, Queen Mary University of London |
| Sadid Hoque | Preventive Neurology Unit, Wolfson Institute of Population Health, Queen Mary University of London |
| Stephanie Mitchell | Northern Care Alliance NHS Trust |
| Stephen Sawcer | University of Cambridge, Department of Clinical Neuroscience, Addenbrookes Hospital, Hills Road, Cambridge, CB22 3TD |
| Tarunya Arun | University Hospitals of Coventry and Warwickshire |
| Tatiana Pogreban | Research and Innovation, Queen's Hospital, BHRUT |
| Terri-Louise Brown | Preventive Neurology Unit, Wolfson Institute of Population Health, Queen Mary University of London |
| Thamanna Begum | Preventive Neurology Unit, Wolfson Institute of Population Health, Queen Mary University of London |
| Veronica Antoine | Northern Care Alliance NHS Trust |
