# Supplementary material for: ADAMS project: a genetic Association study in individuals from Diverse Ancestral backgrounds with Multiple Sclerosis based in the UK
Source: BMJ Open. 2023 May 17;13(5):e071656. doi: 10.1136/bmjopen-2023-071656 (PMC10193065; doi:10.1136/bmjopen-2023-071656)
Supplement: Author's manuscript [file bmjopen-2023-071656.draft_revisions.pdf]

BMJ Open is committed to open peer review. As part of this commitment we make the peer review history of every article we publish publicly available.

When an article is published we post the peer reviewers' comments and the authors' responses online. We also post the versions of the paper that were used during peer review. These are the versions that the peer review comments apply to.

The versions of the paper that follow are the versions that were submitted during the peer review process. They are not the versions of record or the final published versions. They should not be cited or distributed as the published version of this manuscript.

BMJ Open is an open access journal and the full, final, typeset and author-corrected version of record of the manuscript is available on our site with no access controls, subscription charges or pay-per-view fees (http://bmjopen.bmj.com).

If you have any questions on BMJ Open's open peer review process please email info.bmjopen@bmj.com

BMJ Open

Cohort profile: The ADAMS project - a genetic Association study in individuals from Diverse Ancestral backgrounds with Multiple Sclerosis

Journal:	*BMJ Open*
Manuscript ID	bmjopen-2023-071656
Article Type:	Cohort profile
Date Submitted by the Author:	09-Jan-2023
Complete List of Authors:	Jacobs, Benjamin; Wolfson Institute of Population Health, Preventive Neurology Unit<br>Investigators, ADAMS; Wolfson Institute of Population Health, Preventive Neurology Unit<br>Dobson, Ruth; Wolfson Institute of Population Health, Preventive Neurology Unit
Keywords:	Multiple sclerosis < NEUROLOGY, Neurology < INTERNAL MEDICINE, GENETICS

SCHOLARONE™
Manuscripts

I, the Submitting Author has the right to grant and does grant on behalf of all authors of the Work (as defined in the below author licence), an exclusive licence and/or a non-exclusive licence for contributions from authors who are: i) UK Crown employees; ii) where BMJ has agreed a CC-BY licence shall apply, and/or iii) in accordance with the terms applicable for US Federal Government officers or employees acting as part of their official duties; on a worldwide, perpetual, irrevocable, royalty-free basis to BMJ Publishing Group Ltd ("BMJ") its licensees and where the relevant Journal is co-owned by BMJ to the co-owners of the Journal, to publish the Work in this journal and any other BMJ products and to exploit all rights, as set out in our licence.

The Submitting Author accepts and understands that any supply made under these terms is made by BMJ to the Submitting Author unless you are acting as an employee on behalf of your employer or a postgraduate student of an affiliated institution which is paying any applicable article publishing charge ("APC") for Open Access articles. Where the Submitting Author wishes to make the Work available on an Open Access basis (and intends to pay the relevant APC), the terms of reuse of such Open Access shall be governed by a Creative Commons licence – details of these licences and which Creative Commons licence will apply to this Work are set out in our licence referred to above.

Other than as permitted in any relevant BMJ Author's Self Archiving Policies, I confirm this Work has not been accepted for publication elsewhere, is not being considered for publication elsewhere and does not duplicate material already published. I confirm all authors consent to publication of this Work and authorise the granting of this licence.

For peer review only

Title: Cohort profile: The ADAMS project - a genetic Association study in individuals from Diverse Ancestral backgrounds with Multiple Sclerosis

Authors: Benjamin Meir Jacobs, ADAMS investigators, Ruth Dobson

Full author list: Benjamin Meir Jacobs, Luisa Schalk, Joshua Breedon, Catherine Davidson, David Rog, Charles Mein, Eva Wozniak, Charlotte Sellers, Katila George, Thamanna Begum, Michelle Peter, Sadid Hoque, Roxanne Murray, Rashmi Kaimal, Terri-Louise Brown, Eli Silber, Neisha Rule, Lindsey Bezzina, Helen L. Ford, Eve Sacre, Martin O' Malley, Angie Dunne, Emma Tallantyre, Karim Kreft, Emeka Uzochukwu, Stephen Sawcer, Rod Middleton, Katharine Tuite-Dalton, Cord Spilker, Ruth Bellfield, Outi Quinn, Tarunya Arun, Nikos Evangelou, Bruno Gran, David A van Heel, Genes & Health Research Team, Antonio Scalfari, Ghaniah Hassan-Smith, Miriam Mattoscio, Ramya Shamji, Elisa Visentin, Tatiana Pogreban, Waqar Rashid, Gavin Giovannoni, Alastair J Noyce, Huw R Morris, Ruth Dobson

Corresponding author: Ruth Dobson; ruth.dobson@qmul.ac.uk

Affiliations: supplement.

For peer review only

Abstract

Purpose:

Genetic studies of Multiple Sclerosis (MS) susceptibility and severity have focussed on populations of European ancestry. Studying MS genetics in other ancestral groups is necessary to ensure the generalisability of these findings. The ADAMS project aims to gather genetic and phenotypic data on a large cohort of ancestrally-diverse individuals with MS.

Participants:

Adults with self-reported Multiple Sclerosis from diverse (i.e. non-European) ancestral backgrounds. Recruitment is via clinical sites, online (https://app.mantal.co.uk/adams), or the UK MS Register. We are collecting demographic and phenotypic data using a baseline questionnaire and subsequent healthcare record linkage. We are collecting DNA from participants using saliva kits (Oragene-600) and genotyping individuals using the Illumina Global Screening Array version 3.

Findings to date:

We have recruited 682 participants to date (n=446 online, n=55 via sites, n=181 via the UK MS Register). Of this initial cohort, 71.2% of participants are female, with a median age of 44.9 years at recruitment. Over 60% of the cohort are non-White-British, with 23.5% identifying as Asian or Asian British,16.2% as Black, African, Caribbean or Black British, and 20.9% identifying as having mixed or other backgrounds. The median age at first symptom is 28.0 years, and median age at diagnosis is 32.0. 76.8% have relapsing-remitting MS, and 13.5% have secondary progressive MS. The majority (70.3%) are currently receiving or have previously received disease-modifying therapy. 36.8% of our cohort use a walking aid.

Future plans: Recruitment will continue over the next 10 years. Genotyping and genetic data quality control are ongoing. Within the next 3 years we aim to perform initial genetic analyses of susceptibility and severity with a view to replicating the findings from European-ancestry studies. In the long-term, genetic data will be combined with other datasets to further cross-ancestry genetic discoveries.

Registration: Not required

For peer review only

Introduction

The genetic architecture of Multiple Sclerosis (MS) susceptibility in individuals of European ancestry has been extensively assessed[1–5]. Case-control genome wide association studies (GWAS) performed by the International Multiple Sclerosis Genetics Consortium (IMSGC) have discovered 233 independent signals across the genome strongly associated with MS risk, collectively explaining ~39% of heritability[2]. The strongest signals lie within the Major Histocompatibility Complex locus on chromosome 6, with the DRB1*15:01 allele conferring the largest effect of any allele (OR ~3)[6]. More recently, genetic analysis has also shed light on the genetic drivers of disease severity[7].

To date genome-wide screening efforts to identify drivers of MS risk based in populations of non-European ancestry have only involved modest numbers of patients, thus providing limited statistical power[8]. Unsurprisingly none of these studies identified genome-wide significant association, however the single nucelotide polymorphisms (SNPs) associated with MS susceptibility in genetically European populations showed statistically significant evidence of exerting concordant effects on risk in populations of South Asian, African, Hispanic, and ancestrally mixed backgrounds[9–14]. It is inevitable that many of the variants that are relevant in MS will differ in allele frequency between populations, resulting in differences in the power to identify such variants in specific population based association studies, emphasising the value of studying the genetic architecture of MS in different populations[15,16].

Given that patterns of linkage disequilibrium (LD, the correlation between tightly linked variants) also differ greatly between populations, it logically follows that the ability to determine which of the many correlated variants showing association in any given relevant region is driving an association will vary between populations. Those populations with the least extensive LD having the greatest power to finemap that particular association and identify the underlying causal variant driving the association. These efforts to study diverse populations are also expected to improve the generalisability of downstream post-GWAS applications such as polygenic score prediction[8]. In addition to the scientific advances expected from this avenue of research, there is an urgent need for researchers to ensure that trial and study cohorts are diverse and inclusive of the whole population.

Here we report the design and initial cohort phenotype results of a UK-based genetics study of MS risk and severity in individuals of diverse ancestral backgrounds: ADAMS (a genetic Association study in individuals from Diverse Ancestral backgrounds with Multiple Sclerosis). We are prospectively recruiting individuals with MS from diverse ancestries via a web-based platform and via clinical routes with a view to genetic analysis of MS susceptibility and severity. The long-term goal of this project is to combine these data with international datasets to facilitate multi-ancestry genetic analysis of MS.

Cohort description

Recruitment

For peer review only

The ADAMS project is an ongoing genetic cohort study of individuals with MS from diverse ancestral backgrounds living in the UK (https://app.mantal.co.uk/adams). We are recruiting individuals with self-reported Multiple Sclerosis via a bespoke online platform, clinical sites, and the UK MS Register (UKMSR)[17,18]. We are working with networks of primary care practices across the UK where patients have consented for contact regarding research to ensure wide reach of this study. Individuals with a coded diagnosis of MS on their primary care records are contacted with the study information and directed to the study website. Individuals who have previously signed up for the UKMSR can consent to participate in ADAMS from their UKMSR 'home page' at any point. In addition, the UKMSR study team sends regular emails to participants when they are due to complete new online participant-reported outcome measures. These emails contain information about new studies, including ADAMS. Individuals can also sign up via one of our 15 participating clinical sites (full list of participating sites is given in the supplement). Finally, individuals who hear about the study via social media or other public engagement channels can self-refer via the website.

Inclusion and exclusion criteria

Inclusion and exclusion criteria are summarised below:

Inclusion criteria

- Self-reported Multiple Sclerosis (note that diagnoses will be validated against clinical records for a subset of participants)
- Willing and able to give informed consent
- Age > 18
- Willing and able to provide a saliva sample
- Willing to answer baseline demographic questions

Exclusion criteria

- Unable to consent
- Already participating in another study from which we are going to use genomic data (UK Biobank, Genes and Health).

Data collection and genotyping

Once participants have provided informed consent via the dedicated study website, they are directed to a baseline questionnaire. A set of core data elements are collected, including basic demographic details, details about their MS, an address for dispatching the saliva kit, and their NHS number for linkage to their medical records. When participants have completed the demographic questionnaire, they are sent an Oragene-600 saliva kit along with a pre-paid envelope to return to our laboratory. In addition to these data, we are in the process of administering validated questionnaires via our platform to formally assess participants' Expanded Disability Scale Score (EDSS), Multiple Sclerosis Impact Scale (MSIS-29), and the generic EQ5DL, which assess overall health-related quality of life.

The website (https://app.mantal.co.uk/adams) was co-designed with persons with MS. All new data generated as part of this project are stored securely on Queen Mary University of London

(QMUL) servers. Phenotype and genotype data are stored separately and linked by pseudonymous IDs.

Participants are asked to provide consent for researchers to access their medical notes, and for their details to be used to link to national data systems (i.e. NHS Digital). For the initial phase of the study, a subset of participants recruited through BartsMS will have their medical records checked by clinically qualified investigators who will collect a set of core data elements to establish the accuracy of self-reported information submitted by participants. The core data elements set will include: age at symptom onset, age at diagnosis, initial MRI date and findings, initial cerebrospinal fluid (CSF) findings including presence/absence of oligoclonal bands (OCBs), initial diagnostic criteria applied (McDonald version), clinical diagnosis (including MS subtype), history of disease-modifying treatment use, Expanded Disability Scale Score (EDSS) at onset, list of dates and manifestations of clinical relapses, and use of walking aids.

Genotyping is being performed in batches using the Illumina Global Screening Array version 3 with multi-disease booster content (GSA v3 + EAMD) in collaboration with the Genome Centre at QMUL.

Patient and public involvement

Individuals with Multiple Sclerosis from a range of different ethnic backgrounds were involved in this study from its inception and have ongoing input into the management, design, and communications from the study. We work with a 'steering group' of participants who help to run the study.

External datasets & control datasets

We will combine data with large external control datasets for case-control genetic analysis. We will be using data from Genes & Health, a genetic study of ~50,000 individuals of British South Asian ancestry[19], and UK Biobank (UKB)[20]. UKB contains genetic data for ~9000 individuals of South Asian genetic ancestry and ~7000 of African genetic ancestry[21]. Both datasets contain details of significant medical diagnoses of participants, meaning that control-only populations can be derived. These high quality pre-existing genetic datasets will be used as controls for case-control analysis. Additional genetic data from individuals with MS from diverse genetic backgrounds genotyped as part of other studies will be obtained via collaborators.

Target recruitment and power calculations

We have performed power calculations to assess the expected number of cases required to replicate European MS susceptibility alleles (i.e. find association at a nominal, unadjusted P value of <0.05). We calculated power for each of the 233 European MS susceptibility SNPs by assuming an equivalent marginal effect size across ancestries, taking allele frequency differences between ancestries into account. (https://github.com/benjacobs123456/popPoweR). We used discovery-stage summary statistics from the International Multiple Sclerosis Genetics Consortium (IMSGC) GWAS to determine European-ancestry effect sizes[2].

For peer review only - http://bmjopen.bmj.com/site/about/guidelines.xhtml

These calculations demonstrate that recruiting ~300 cases within each ancestral cluster will yield reasonable power to replicate the European susceptibility alleles. We will have power to detect approximately 193/289 and 176/289 of the European top hits in South Asian and African-ancestry individuals respectively with a case sample size of n=300. Mean power across all susceptibility variants in the African population is estimated at 61% and at 67% in the South Asian population. With n=500 in each ancestral group, mean power increases to 69% (African ancestry) and 75% (South Asian ancestry). As the majority of MHC associations are of large magnitude (~OR 3) and genome-wide significant effects are in the order of OR 1.2 – 1.3, we anticipate good power to prove replication at most of the ~233 genome-wide significant loci within this ancestrally-diverse cohort.

Analytic plan

We will conduct genetic analysis of MS susceptibility and severity within each ancestral cluster. Ancestral groups will be defined by using principal component analysis and global ancestry inference. Genome-wide association testing will be performed using mixed logistic models. We will perform a meta-analysis on these results with GWAS summary statistics from European-ancestry populations using random and fixed-effect meta-analysis. A variety of post-GWAS analyses will be conducted, including cross-ancestry genetic correlation, fine mapping, polygenic risk score profiling, and mendelian randomisation.

Findings to date

As of January 3rd 2023, we have recruited 682 participants within the first year of the study (n=446 via the website, n=55 in person at clinical sites, n=181 via the UK MS Register). Of this initial cohort, 71.2% of participants are female, with a median age of 44.9 years at recruitment. Over 60% of the cohort are non-White-British, with 23.5% identifying as Asian or Asian British,16.2% as Black, African, Caribbean or Black British, 20.9% identifying as having mixed or other backgrounds, a sizeable proportion (10.7%) preferring to not disclose their ethnic background, and 28.7% identifying as White. A substantial proportion were born outside the UK (23.1%).

We have gathered self-reported data from participants to facilitate genetic analyses of phenotypes. The median age at first symptom is 28.0, and the median age at diagnosis is 32.0. 76.8% have relapsing-remitting MS, and 13.5% have secondary progressive MS. The majority (70.3%) are currently receiving and/or have previously received disease-modifying therapy. 43.0% of our cohort stated that they are unlimited in their mobility, and 36.8% use a walking aid at time of recruitment (approximate Expanded Disability Status Scale score of 6 or more).

The online questionnaire data also provides an insight into risk factors for MS. 20.0% of participants reported being overweight during adolescence, 16.8% report a family history of MS, 47.2% report ever having smoked, and 15.3% report having had glandular fever.

The average age at first reported symptom tended to be earlier in individuals who identified as South Asian (mean 26.4 [8.3]) compared with self-reported Black individuals (mean 30.6 [12.1],

For peer review only

p=0.02) and White individuals (mean 31.6 [10.4]; p<0.0001). We observed a similar pattern for age at self-reported diagnosis, with Black (mean 32.9 [12.1], p=0.04) and Asian (mean 29.7 [8.5], p<0.0001) individuals reporting an earlier age at diagnosis than White individuals (mean 36.5 [10.1]). The gender balance of the recruited cohort also differs between ethnic groups (p=0.03), with a higher proportion of men in the self-reported Asian cohort (39% male) compared with the White (25.6% male) and Black cohorts (22.4% male). These differences in demographic characteristics, although based on a small cohort so far, are consistent with previous findings[22].

Strengths and limitations

Strengths

- ADAMS will be a large, ancestrally-diverse, genotyped cohort of individuals with MS, which will help to extend genetic analysis of MS to people from non-European ancestry.
- Online recruitment facilitates rapid scaling of the study, with minimal imposition on participants.
- ADAMS also recruits directly from multiple UK clinical sites, where participants can sign up via custom website links or ‘in-person’ using paper forms.
- The current recruitment rate demonstrates the feasibility of reaching our recruitment targets.

Limitations

- Relying on online self-report for MS diagnosis and phenotype information may not be as accurate as clinician-determined measures.
- Online recruitment is likely to introduce various biases, for instance it is likely to discourage older and more disabled individuals from signing up.
- This is a case-only cohort. The accuracy of case-control analyses relies on the abundance of ancestrally-similar controls in UK Biobank and other control datasets.
- Much larger sample sizes are required to make novel genetic discoveries.

Conclusions

ADAMS (https://app.mantal.co.uk/adams) is a genetic cohort study aiming to determine the genetic basis of Multiple Sclerosis risk and severity in individuals from non-European ancestral backgrounds living in the UK. Recruitment and genotyping are ongoing. Individuals with MS or potential collaborators can contact the study team via adams_study@qmul.ac.uk.

Collaboration & data sharing

GWAS summary statistics will be publicly-available. Individual-level genotypes without identifying information will be shared with *bona fide* researchers on application via the European Genome-Phenome Archive (EGA). We are seeking collaborators for data sharing and/or widening our recruitment network. Please contact the lead author for further information.

Ethical approval

For peer review only - http://bmjopen.bmj.com/site/about/guidelines.xhtml

This study and its amendments have been approved by the London - South East Research Ethics Committee (REC reference 21/PR/1289).

Funding

This study is funded by an Medical Research Council (MRC) Clinical Research Training Fellowship (CRTF) jointly supported by the UK MS Society (BMJ; grant reference MR/V028766/1), AIMS2CURE (grant reference N/A), and Barts Charity.

Competing interests

The authors have no relevant competing interests to declare.

Author contributions

A full list of contributing authors and their contributions can be found in the supplement.

BJ and RD designed the study. BJ, RD, HRM, GG, ES, and AJN were involved in the conception of the study. BJ and RD have ultimate oversight over the study. BJ wrote the first draft of the manuscript. BJ and LS are reponsible for the day-to-day management of the study. BJ analysed the data. All authors are involved in data acquisition and/or recruitment. All authors were involved in drafting and editing the manuscript, have given final approval for submission, and accept accountability for the work reported.

References

1. International Multiple Sclerosis Genetics Consortium (IMSGC) *et al.* Analysis of immune-related loci identifies 48 new susceptibility variants for multiple sclerosis. *Nat. Genet.* **45**, 1353–1360 (2013).
2. International Multiple Sclerosis Genetics Consortium. Multiple sclerosis genomic map implicates peripheral immune cells and microglia in susceptibility. *Science* **365**, (2019).
3. Sawcer, S. *et al.* A high-density screen for linkage in multiple sclerosis. *Am. J. Hum. Genet.* **77**, 454–467 (2005).
4. International Multiple Sclerosis Genetics Consortium. Electronic address: chris.cotsapas@yale.edu & International Multiple Sclerosis Genetics Consortium. Low-Frequency and Rare-Coding Variation Contributes to Multiple Sclerosis Risk. *Cell* **178**, 262 (2019).
5. International Multiple Sclerosis Genetics Consortium *et al.* Genetic risk and a primary role

For peer review only

for cell-mediated immune mechanisms in multiple sclerosis. *Nature* **476**, 214–219 (2011).

6. Moutsianas, L. *et al.* Class II HLA interactions modulate genetic risk for multiple sclerosis. *Nat. Genet.* **47**, 1107–1113 (2015).
7. Baranzini, S., Sawcer, S., International Multiple Sclerosis Genetics Consortium & MultipleMS Consortium. Genetic analysis of multiple sclerosis severity identifies a novel locus and implicates CNS resilience as a major determinant of outcome. *Research Square* (2022) doi:10.21203/rs.3.rs-1723574/v1.
8. Jacobs, B. M. *et al.* Towards a global view of multiple sclerosis genetics. *Nat. Rev. Neurol.* (2022) doi:10.1038/s41582-022-00704-y.
9. Pandit, L. *et al.* HLA associations in South Asian multiple sclerosis. *Mult. Scler.* **22**, 19–24 (2016).
10. Pandit, L. *et al.* Evaluation of the established non-MHC multiple sclerosis loci in an Indian population. *Mult. Scler.* **17**, 139–143 (2011).
11. Beecham, A. H. *et al.* The genetic diversity of multiple sclerosis risk among Hispanic and African American populations living in the United States. *Mult. Scler.* **26**, 1329–1339 (2020).
12. Isobe, N. *et al.* An ImmunoChip study of multiple sclerosis risk in African Americans. *Brain* **138**, 1518–1530 (2015).
13. Isobe, N. *et al.* Genetic risk variants in African Americans with multiple sclerosis. *Neurology* **81**, 219–227 (2013).
14. Chi, C. *et al.* Admixture mapping reveals evidence of differential multiple sclerosis risk by genetic ancestry. *PLoS Genet.* **15**, e1007808 (2019).
15. Steri, M. *et al.* Overexpression of the Cytokine BAFF and Autoimmunity Risk. *N. Engl. J. Med.* **376**, 1615–1626 (2017).
16. Beecham, A. H. *et al.* Ancestral risk modification for multiple sclerosis susceptibility detected across the Major Histocompatibility Complex in a multi-ethnic population. *PLoS One* **17**, e0279132 (2022).

For peer review only

17. Middleton, R. M. *et al.* Validating the portal population of the United Kingdom Multiple Sclerosis Register. *Mult. Scler. Relat. Disord.* **24**, 3–10 (2018).

18. Ford, D. V. *et al.* The feasibility of collecting information from people with Multiple Sclerosis for the UK MS Register via a web portal: characterising a cohort of people with MS. *BMC Med. Inform. Decis. Mak.* **12**, 73 (2012).

19. Finer, S. *et al.* Cohort Profile: East London Genes & Health (ELGH), a community-based population genomics and health study in British Bangladeshi and British Pakistani people. *Int. J. Epidemiol.* **49**, 20–21i (2020).

20. Bycroft, C. *et al.* The UK Biobank resource with deep phenotyping and genomic data. *Nature* **562**, 203–209 (2018).

21. Privé, F. Using the UK Biobank as a global reference of worldwide populations: application to measuring ancestry diversity from GWAS summary statistics. *Bioinformatics* **38**, 3477–3480 (2022).

22. Jacobs, B. M. *et al.* Modifiable Risk Factors for Multiple Sclerosis are Consistent Across Diverse Ethnic Backgrounds: A Nested Case-Control Study in a UK Population-Based Cohort. (2022) doi:10.2139/ssrn.4237729.

For peer review only

Full name	Primary affiliation
Ruth Dobson	Preventive Neurology Unit, Wolfson Institute of Population Health, Qu
Benjamin M Jacobs	Preventive Neurology Unit, Wolfson Institute of Population Health, Qu
Luisa Schalk	Preventive Neurology Unit, Wolfson Institute of Population Health, Qu
Huw Morris	Department of Clinical and Movement Neuroscience, UCL Queen Sc
Gavin Giovannoni	Preventive Neurology Unit, Wolfson Institute of Population Health, Qu
Alastair J Noyce	Preventive Neurology Unit, Wolfson Institute of Population Health, Qu
Eli Silber	Kings College Hospital and Lewisham and Greenwich NHS Trusts
Joshua Breedon	Preventive Neurology Unit, Wolfson Institute of Population Health, Qu
Katila George	Preventive Neurology Unit, Wolfson Institute of Population Health, Qu
Thamanna Begum	Preventive Neurology Unit, Wolfson Institute of Population Health, Qu
Charlotte Sellers	Preventive Neurology Unit, Wolfson Institute of Population Health, Qu
Stephanie Mitchell	Northern Care Alliance NHS Trust
Nimisha Vinod	Northern Care Alliance NHS Trust
Veronica Antoine	Northern Care Alliance NHS Trust
Jade Harris	Northern Care Alliance NHS Trust
Judith Brooke	Northern Care Alliance NHS Trust
Ramya Shamji	Research and Innovation, Queen's Hospital, BHRUT
David Rog	Manchester Centre for Clinical Neurosciences, Northern Care Allianc
Helen L. Ford	Leeds Centre for Neurosciences, Leeds Teaching Hospitals NHS Tru
Elisa Visentin	Research and Innovation, Queen's Hospital, BHRUT
Neisha Rhule	Queen Elizabeth Hospital (Lewisham and Greenwich NHS Trust)
Bruno Gran	Department of Neurology, Nottingham University Hospitals NHS Trus
Eve Sacre	Leeds Centre for Neurosciences, Leeds teaching Hospitals NHS Tru
Martin O'Malley	Leeds Centre for Neurosciences, Leeds teaching Hospitals NHS Tru
Angie Dunne	Leeds Centre for Neurosciences, Leeds teaching Hospitals NHS Tru
Miriam Mattoscio	Department of neuroscience, Queen's Hospital, BHRUT NHS Trust
Tatiana Pogreban	Research and Innovation, Queen's Hospital, BHRUT
Elizabeth Lindsey Bezzina	
Stephen Sawcer	University of Cambridge, Department of Clinical Neuroscience, Adde
Cord Spilker	Bradford Teaching Hospital Foundation Trust
Karim L. Kreft	Department of Neurology, University Hospital of Wales, Cardiff
Rod Middleton	Population Data Science, Swansea University Medical School, Swan
Tarunya Arun	
Emma Tallantyre	1. Division of Psychological Medicine and Clinical Neurosciences, Ca
Emeka Uzochukwu	Division of Psychological Medicine and Clinical Neurosciences, Cardi
Charles A Mein	Barts and the London Genome Centre, Queen Mary University of Lor
Eva Wozniak	Barts and the London Genome Centre, Queen Mary University of Lor
Roxanne Murray	Preventive Neurology Unit, Wolfson Institute of Population Health, Qu
Michelle Peter	Preventive Neurology Unit, Wolfson Institute of Population Health, Qu
Sadid Hoque	Preventive Neurology Unit, Wolfson Institute of Population Health, Qu
Rashmi Kaimal	Preventive Neurology Unit, Wolfson Institute of Population Health, Qu
Terri-Louise Brown	Preventive Neurology Unit, Wolfson Institute of Population Health, Qu

For peer review only

For peer review only

BMJ Open

Cohort profile: The ADAMS project - a genetic Association study in individuals from Diverse Ancestral backgrounds with Multiple Sclerosis based in the United Kingdom

Journal:	*BMJ Open*
Manuscript ID	bmjopen-2023-071656.R1
Article Type:	Cohort profile
Date Submitted by the Author:	31-Mar-2023
Complete List of Authors:	Jacobs, Benjamin; Wolfson Institute of Population Health, Preventive Neurology Unit<br>Investigators, ADAMS; Wolfson Institute of Population Health, Preventive Neurology Unit<br>Dobson, Ruth; Wolfson Institute of Population Health, Preventive Neurology Unit
**Primary Subject Heading**:	Neurology
Secondary Subject Heading:	Epidemiology, Neurology
Keywords:	Multiple sclerosis < NEUROLOGY, Neurology < INTERNAL MEDICINE, GENETICS

SCHOLARONE™
Manuscripts

I, the Submitting Author has the right to grant and does grant on behalf of all authors of the Work (as defined in the below author licence), an exclusive licence and/or a non-exclusive licence for contributions from authors who are: i) UK Crown employees; ii) where BMJ has agreed a CC-BY licence shall apply, and/or iii) in accordance with the terms applicable for US Federal Government officers or employees acting as part of their official duties; on a worldwide, perpetual, irrevocable, royalty-free basis to BMJ Publishing Group Ltd ("BMJ") its licensees and where the relevant Journal is co-owned by BMJ to the co-owners of the Journal, to publish the Work in this journal and any other BMJ products and to exploit all rights, as set out in our licence.

The Submitting Author accepts and understands that any supply made under these terms is made by BMJ to the Submitting Author unless you are acting as an employee on behalf of your employer or a postgraduate student of an affiliated institution which is paying any applicable article publishing charge ("APC") for Open Access articles. Where the Submitting Author wishes to make the Work available on an Open Access basis (and intends to pay the relevant APC), the terms of reuse of such Open Access shall be governed by a Creative Commons licence – details of these licences and which Creative Commons licence will apply to this Work are set out in our licence referred to above.

Other than as permitted in any relevant BMJ Author's Self Archiving Policies, I confirm this Work has not been accepted for publication elsewhere, is not being considered for publication elsewhere and does not duplicate material already published. I confirm all authors consent to publication of this Work and authorise the granting of this licence.

For peer review only

Title: Cohort profile: The ADAMS project - a genetic Association study in individuals from Diverse Ancestral backgrounds with Multiple Sclerosis based in the United Kingdom

Authors: Benjamin Meir Jacobs, ADAMS investigators, Ruth Dobson

Full author list: Benjamin Meir Jacobs, Luisa Schalk, Joshua Breedon, Catherine Davidson, David Rog, Charles Mein, Eva Wozniak, Charlotte Sellers, Katila George, Thamanna Begum, Michelle Peter, Sadid Hoque, Roxanne Murray, Rashmi Kaimal, Terri-Louise Brown, Eli Silber, Neisha Rhule, Lindsey Bezzina, Helen L. Ford, Eve Sacre, Martin O' Malley, Angie Dunne, Emma Tallantyre, Karim Kreft, Emeka Uzochukwu, Stephen Sawcer, Rod Middleton, Katharine Tuite-Dalton, Cord Spilker, Ruth Bellfield, Outi Quinn, Tarunya Arun, Nikos Evangelou, Bruno Gran, David A van Heel, Genes & Health Research Team, Antonio Scalfari, Ghaniah Hassan-Smith, Miriam Mattoscio, Ramya Shamji, Elisa Visentin, Tatiana Pogreban, Waqar Rashid, Gavin Giovannoni, Alastair J Noyce, Huw R Morris, Ruth Dobson

Corresponding author: Ruth Dobson; ruth.dobson@qmul.ac.uk

Affiliations: supplement.

For peer review only

Abstract

Purpose:

Genetic studies of Multiple Sclerosis (MS) susceptibility and severity have focussed on populations of European ancestry. Studying MS genetics in other ancestral groups is necessary to determine the generalisability of these findings. The genetic Association study in individuals from Diverse Ancestral backgrounds with Multiple Sclerosis (ADAMS) project aims to gather genetic and phenotypic data on a large cohort of ancestrally-diverse individuals with MS living in the UK.

Participants:

Adults with self-reported Multiple Sclerosis from diverse ancestral backgrounds. Recruitment is via clinical sites, online (https://app.mantal.co.uk/adams), or the UK MS Register. We are collecting demographic and phenotypic data using a baseline questionnaire and subsequent healthcare record linkage. We are collecting DNA from participants using saliva kits (Oragene-600) and genotyping using the Illumina Global Screening Array version 3.

Findings to date:

As of January 3rd 2023, we have recruited 682 participants (n=446 online, n=55 via sites, n=181 via the UK MS Register). Of this initial cohort, 71.2% of participants are female, with a median age of 44.9 years at recruitment. Over 60% of the cohort are non-White-British, with 23.5% identifying as Asian or Asian British,16.2% as Black, African, Caribbean or Black British, and 20.9% identifying as having mixed or other backgrounds. The median age at first symptom is 28.0 years, and median age at diagnosis is 32.0. 76.8% have relapsing-remitting MS, and 13.5% have secondary progressive MS.

Future plans: Recruitment will continue over the next 10 years. Genotyping and genetic data quality control are ongoing. Within the next 3 years we aim to perform initial genetic analyses of susceptibility and severity with a view to replicating the findings from European-ancestry studies. In the long-term, genetic data will be combined with other datasets to further cross-ancestry genetic discoveries.

Registration: Not required

For peer review only

Strengths and limitations

Strengths

- ADAMS will be a large, ancestrally-diverse, genotyped cohort of individuals with MS, which will help to extend genetic analysis of MS to people from non-European ancestral backgrounds. Online recruitment facilitates rapid scaling of the study, with minimal imposition on participants. The current recruitment rate demonstrates the feasibility of reaching our recruitment targets.

Limitations

- Online recruitment is likely to introduce various biases, for instance it may discourage older and more disabled individuals from signing up.
- Relying on online self-report for MS diagnosis and phenotype information may not be as accurate as clinician-determined measures – there is a small risk that people without clinically-definite MS may sign up for the study.
- This is a case-only cohort. The accuracy of case-control analyses relies on the abundance of ancestrally-similar controls in UK Biobank and other control datasets.
- Much larger sample sizes are required to make novel genetic discoveries.

For peer review only

Introduction

The genetic architecture of Multiple Sclerosis (MS) susceptibility in individuals of European ancestry has been extensively assessed[1–5]. Case-control genome wide association studies (GWAS) performed by the International Multiple Sclerosis Genetics Consortium (IMSGC) have discovered 233 independent signals across the genome strongly associated with MS risk, collectively explaining ~39% of heritability[2]. The strongest signals lie within the Major Histocompatibility Complex locus on chromosome 6, with the DRB1*15:01 allele conferring the largest effect of any allele (OR ~3)[6]. More recently, genetic analysis has also shed light on the genetic drivers of disease severity[7].

To date genome-wide screening efforts to identify determinants of MS risk in populations of non-European ancestry have only involved modest numbers of participants, thus providing limited statistical power[8]. Unsurprisingly none of these studies has identified new genome-wide significant associations, however the single nucelotide polymorphisms (SNPs) associated with MS susceptibility in genetically-European populations have shown concordant effects on risk in populations of South Asian, African, Hispanic, and ancestrally mixed backgrounds[9–16]. It is inevitable that many of the variants of relevance in MS will differ in allele frequency between populations. This will potentially result in differences in power to identify such variants in specific populations, emphasising the value of studying the genetic architecture of MS in diverse cohorts[16,17].

Patterns of linkage disequilibrium (LD, the correlation between genetic variants) also differ greatly between ancestral populations. Due to LD, it can be challenging to identify the underlying causal variant/s responsible for a region of genetic association. Populations with less extensive LD – such as populations of African ancestry - afford greater power for fine-mapping as the number of variants which could plausibly account for any given GWAS signal tends to be lower[8,18]. The complementary patterns of LD between ancestries can also be leveraged to further improve fine-mapping.

Genetic studies of diverse populations are also expected to improve the generalisability of downstream post-GWAS applications such as polygenic score prediction and mendelian randomisation[8]. In addition to the scientific advances expected from this avenue of research, broadening participation in medical research is valuable both in itself and for its instrumental societal impact[19].

Here we report the design and initial cohort phenotype results of a UK-based genetic study of MS risk and severity in individuals of diverse ancestral backgrounds: ADAMS (a genetic Association study in individuals from Diverse Ancestral backgrounds with Multiple Sclerosis). We are prospectively recruiting individuals with MS from diverse ancestries via a web-based platform and via clinical routes with a view to performing genetic analysis of MS susceptibility and severity. The long-term goal of this project is to combine these data with international datasets to facilitate multi-ancestry genetic analysis of MS.

Cohort description

For peer review only

Recruitment

The ADAMS project is an ongoing genetic cohort study of individuals with MS from diverse ancestral backgrounds living in the UK (https://app.mantal.co.uk/adams). We are recruiting individuals with self-reported Multiple Sclerosis via a bespoke online platform, clinical sites, and the UK MS Register (UKMSR)[20,21]. We are working with networks of primary care practices across the UK where patients have consented for contact regarding research to ensure wide reach of this study. Individuals with a coded diagnosis of MS on their primary care records are contacted with the study information and directed to the study website. Individuals who have previously signed up for the UKMSR can consent to participate in ADAMS from their UKMSR 'home page' at any point. In addition, the UKMSR study team sends regular emails to participants when they are due to complete new online participant-reported outcome measures. These emails contain information about new studies, including ADAMS. Individuals can also sign up via one of our 15 participating clinical sites (full list of participating sites is given in the supplement). Finally, individuals who hear about the study via social media or other public engagement channels can self-refer via the website.

Inclusion and exclusion criteria

This study is focussed on people with MS from diverse ancestral backgrounds, i.e. people with recent non-European ancestry. However, self-reported ethnicity or ancestry is not a strict inclusion/exclusion criterion. Our rationale for this is that our study aims to be as inclusive as possible, and self-reported ethnicity is a relatively crude and poor proxy for genetic ancestry. We are therefore recruiting from a diverse audience and will infer genetic ancestry from genotyping data as part of the data analysis pipeline.

Inclusion and exclusion criteria are summarised below:

<u>Inclusion criteria</u>

- Self-reported Multiple Sclerosis (diagnosis will be validated against clinical records for a subset of participants)
- Willing and able to give informed consent
- Age > 18
- Willing and able to provide a saliva sample
- Willing to answer baseline survey questions

<u>Exclusion criteria</u>

- Unable to consent
- Already participating in another study from which we are going to use genomic data (UK Biobank, Genes and Health).

Data collection and genotyping

Once participants have provided informed consent via the dedicated study website, they are directed to a baseline questionnaire. A set of core data elements are collected, including basic demographic details, details about their MS, an address for dispatching the saliva kit, and their NHS number for linkage to their medical records. When participants have completed the demographic questionnaire, they are sent an Oragene-600 saliva kit along with a pre-paid

For peer review only

envelope to return to our laboratory. In addition to these data, we are in the process of administering validated questionnaires via our platform to formally assess participants' Expanded Disability Scale Score (EDSS), Multiple Sclerosis Impact Scale (MSIS-29), and the EQ-5D-5L, which assess overall health-related quality of life. The current consent form and participant information sheet can be downloaded from https://app.mantal.co.uk/adams/consent?hl=GB.

The website (https://app.mantal.co.uk/adams) was co-designed with persons with MS. All new data generated as part of this project are stored securely on Queen Mary University of London (QMUL) servers. Phenotype and genotype data are stored separately and linked by pseudonymous IDs.

Participants are asked to provide consent for researchers to access their medical notes, and for their details to be used to link to national data systems (i.e. via NHS Digital). For the initial phase of the study, a subset of participants recruited through the primary NHS site (Barts Health NHS Trust) will have their medical records checked by clinically qualified investigators who will collect a set of core data elements to establish the accuracy of self-reported information submitted by participants. The core data elements set will include: age at symptom onset, age at diagnosis, initial MRI date and findings, initial cerebrospinal fluid (CSF) findings including presence/absence of oligoclonal bands (OCBs), initial diagnostic criteria applied (McDonald version), clinical diagnosis (including MS subtype), history of disease-modifying treatment use, Expanded Disability Scale Score (EDSS) at onset, list of dates and manifestations of clinical relapses, and use of walking aids.

Genotyping is being performed in batches using the Illumina Global Screening Array version 3 with multi-disease booster content (GSA v3 + EAMD) in collaboration with the Genome Centre at QMUL[15,22,23].

Patient and public involvement

Individuals with Multiple Sclerosis from a range of different ethnic backgrounds were involved in this study from its inception and have ongoing input into the management, design, and communications from the study. We are working with a group of participants who help to run the study.

External datasets & control datasets

We will combine data with large external control datasets for case-control genetic analysis. We will be using data from Genes & Health, a genetic study of ~50,000 individuals of British South Asian ancestry[23], and UK Biobank (UKB)[24]. UKB contains genetic data for ~9000 individuals of South Asian genetic ancestry and ~7000 of African genetic ancestry[25]. Both datasets contain details of significant medical diagnoses of participants, meaning that control-only populations can be derived. These high quality pre-existing genetic datasets will be used as controls for case-control analysis. Additional genetic data from individuals with MS from diverse genetic backgrounds genotyped as part of other studies will be obtained via collaborators.

For peer review only

Target recruitment and power calculations

We have performed power calculations to assess the expected number of cases required to replicate European MS susceptibility alleles (i.e. find association at a nominal, unadjusted P value of <0.05). We calculated power for each of the 233 European MS susceptibility SNPs by assuming an equivalent marginal effect size across ancestries, taking allele frequency differences between ancestries into account. (https://github.com/benjacobs123456/popPoweR). We used discovery-stage summary statistics from the International Multiple Sclerosis Genetics Consortium (IMSGC) GWAS to determine European-ancestry effect sizes[2].

These calculations demonstrate that recruiting ~300 cases within each ancestral cluster will yield reasonable power to replicate the European susceptibility alleles. We will have power to detect approximately 193/289 and 176/289 of the European risk alleles in South Asian and African-ancestry individuals respectively with a case sample size of n=300. Mean power across all susceptibility variants in the African population is estimated at 61% and at 67% in the South Asian population. With n=500 in each ancestral group, mean power increases to 69% (African ancestry) and 75% (South Asian ancestry). As the majority of MHC associations are of large magnitude (~OR 3) and genome-wide significant effects are in the order of OR 1.2 – 1.3, we anticipate good power to prove replication at most of the ~233 genome-wide significant loci within this ancestrally-diverse cohort. We will not have statistical power to discover novel associations through this cohort with the current recruitment target. Furthermore, it is important to note that these power calculations are rough estimates – and may be overestimates - as we do not yet know the precise ancestral composition of the cohort, and there is significant genetic diversity within these broad ancestral clusters.

Analytic plan

We will conduct genetic analysis of MS susceptibility and severity within each ancestral cluster. Ancestral groups will be defined using principal component analysis and global ancestry inference. Genome-wide association testing will be performed using mixed logistic or linear models (for case-control and continuous phenotypes respectively). We will perform a meta-analysis on these results with GWAS summary statistics from European-ancestry populations using random and fixed-effect meta-analysis. A variety of post-GWAS analyses will be conducted, including cross-ancestry genetic correlation, fine mapping, polygenic risk score profiling, and mendelian randomisation.

Findings to date

Recruitment started in November 2021 and is currently expected to run until August 2031 pending long-term funding. As of January 3rd 2023, we have recruited 682 participants (n=446 via the website, n=55 in person at clinical sites, n=181 via the UK MS Register). Of this initial cohort, 71.2% of participants are female, with a median age of 44.9 years at recruitment [Interquartile range; IQR 18.3]. Over 60% of the cohort are non-White-British, with 23.5% identifying as Asian or Asian British,16.2% as Black, African, Caribbean or Black British, and 20.9% identifying as having mixed or other backgrounds. A sizeable proportion (10.7%) prefer to not disclose their ethnic background, and 28.7% identify as White. For the substantial

For peer review only - http://bmjopen.bmj.com/site/about/guidelines.xhtml

proportion of the cohort born outside the UK (23.1%), the median age at migration was 18.0 [IQR 20.0].

We have gathered self-reported data from participants to facilitate genetic analyses of phenotypes. The median age at first symptom is 28.0 [IQR 15.0], and the median age at diagnosis 32.0 [IQR 14.0]. 76.8% have relapsing-remitting MS, and 13.5% have secondary progressive MS. The majority (70.3%) are currently receiving and/or have previously received disease-modifying therapy. 43.0% of our cohort stated that they are unlimited in their mobility, and 36.8% report using a walking aid at time of recruitment (approximate Expanded Disability Status Scale score of at least 6.0).

Questionnaire data also provides an insight into risk factors for MS. 20.0% of participants reported being overweight during adolescence, 16.8% report a family history of MS, 47.2% report ever having smoked, and 15.3% report having had glandular fever.

The average age at first reported symptom tended to be earlier in individuals who identified as South Asian (mean 26.4 [SD 8.3]) compared with self-reported Black individuals (mean 30.6 [SD 12.1], $p=0.02$) and White individuals (mean 31.6 [SD 10.4]; $p<0.0001$). We observed a similar pattern for age at self-reported diagnosis, with Black (mean 32.9 [SD 12.1], $p=0.04$) and Asian (mean 29.7 [SD 8.5], $p<0.0001$) individuals reporting an earlier age at diagnosis than White individuals (mean 36.5 [SD 10.1]). The gender balance of the recruited cohort also differs between ethnic groups ($p=0.03$), with a higher proportion of men in the self-reported Asian cohort (39% male) compared with the White (25.6% male) and Black cohorts (22.4% male). These differences in demographic characteristics, although based on a small cohort so far, are consistent with previous findings[26].

Conclusions

ADAMS (https://app.mantal.co.uk/adams) is a genetic cohort study aiming to determine the genetic basis of Multiple Sclerosis risk and severity in individuals from non-European ancestral backgrounds living in the UK. Recruitment and genotyping are ongoing. Individuals with MS or potential collaborators can contact the study team via adams_study@qmul.ac.uk.

Ethical approval

This study and its amendments have been approved by the London - South East Research Ethics Committee (REC reference 21/PR/1289).

Funding

This study is funded by an Medical Research Council (MRC) Clinical Research Training Fellowship (CRTF) jointly supported by the UK MS Society (BMJ; grant reference MR/V028766/1), AIMS2CURE (grant reference N/A), and Barts Charity. This work is being carried out at the Preventive Neurology Unit at Queen Mary University of London which is partly funded by Barts Charity.

For peer review only

Competing interests
The authors have no relevant competing interests to declare.

Author contributions

BJ and RD designed the study and have ultimate oversight over the study. BJ wrote the first draft of the manuscript. BJ is responsible for the day-to-day management of the study. BJ analysed the data.

Acknowledgements
A full list of contributing authors and their contributions can be found in the supplement.
BJ, RD, HRM, GG, ES, and AJN were involved in the conception of the study. BJ and RD have ultimate oversight over the study. BJ wrote the first draft of the manuscript. BJ and LS are responsible for the day-to-day management of the study. BJ analysed the data. All authors are involved in data acquisition and/or recruitment. All authors were involved in drafting and editing the manuscript, have given final approval for submission, and accept accountability for the work reported.

Data sharing statement
Genetic association summary statistics will be made publicly available on completion of the pre-planned analyses. Genetic data for participants who sign up via the UK MS Register will be fed back into their secure data safe haven and will be available to *bona fide* researchers on request at https://ukmsregister.org/. Pseudonymised individual-level genotype data will be made available via the European Genome-Phenome Archive (EGA). We are seeking collaborators for data sharing and/or widening our recruitment network. Please contact the lead author or the generic study email address (adams_study@qmul.ac.uk) for further information.

References

1. International Multiple Sclerosis Genetics Consortium (IMSGC) *et al.* Analysis of immune-related loci identifies 48 new susceptibility variants for multiple sclerosis. *Nat. Genet.* **45**, 1353–1360 (2013).
2. International Multiple Sclerosis Genetics Consortium. Multiple sclerosis genomic map implicates peripheral immune cells and microglia in susceptibility. *Science* **365**, (2019).
3. Sawcer, S. *et al.* A high-density screen for linkage in multiple sclerosis. *Am. J. Hum. Genet.* **77**, 454–467 (2005).

For peer review only - http://bmjopen.bmj.com/site/about/guidelines.xhtml

4. International Multiple Sclerosis Genetics Consortium. Electronic address: chris.cotsapas@yale.edu & International Multiple Sclerosis Genetics Consortium. Low-Frequency and Rare-Coding Variation Contributes to Multiple Sclerosis Risk. *Cell* **178**, 262 (2019).
5. International Multiple Sclerosis Genetics Consortium *et al.* Genetic risk and a primary role for cell-mediated immune mechanisms in multiple sclerosis. *Nature* **476**, 214–219 (2011).
6. Moutsianas, L. *et al.* Class II HLA interactions modulate genetic risk for multiple sclerosis. *Nat. Genet.* **47**, 1107–1113 (2015).
7. Baranzini, S., Sawcer, S., International Multiple Sclerosis Genetics Consortium & MultipleMS Consortium. Genetic analysis of multiple sclerosis severity identifies a novel locus and implicates CNS resilience as a major determinant of outcome. *Research Square* (2022) doi:10.21203/rs.3.rs-1723574/v1.
8. Jacobs, B. M. *et al.* Towards a global view of multiple sclerosis genetics. *Nat. Rev. Neurol.* (2022) doi:10.1038/s41582-022-00704-y.
9. Pandit, L. *et al.* HLA associations in South Asian multiple sclerosis. *Mult. Scler.* **22**, 19–24 (2016).
10. Pandit, L. *et al.* Evaluation of the established non-MHC multiple sclerosis loci in an Indian population. *Mult. Scler.* **17**, 139–143 (2011).
11. Beecham, A. H. *et al.* The genetic diversity of multiple sclerosis risk among Hispanic and African American populations living in the United States. *Mult. Scler.* **26**, 1329–1339 (2020).
12. Isobe, N. *et al.* An ImmunoChip study of multiple sclerosis risk in African Americans. *Brain* **138**, 1518–1530 (2015).
13. Isobe, N. *et al.* Genetic risk variants in African Americans with multiple sclerosis. *Neurology* **81**, 219–227 (2013).
14. Chi, C. *et al.* Admixture mapping reveals evidence of differential multiple sclerosis risk by genetic ancestry. *PLoS Genet.* **15**, e1007808 (2019).

For peer review only

15. Breedon, J. R. *et al.* Polygenic risk score prediction of multiple sclerosis in individuals of South Asian ancestry. *Brain Commun.* (2023) doi:10.1093/braincomms/fcad041.

16. Beecham, A. H. *et al.* Ancestral risk modification for multiple sclerosis susceptibility detected across the Major Histocompatibility Complex in a multi-ethnic population. *PLoS One* **17**, e0279132 (2022).

17. Steri, M. *et al.* Overexpression of the Cytokine BAFF and Autoimmunity Risk. *N. Engl. J. Med.* **376**, 1615–1626 (2017).

18. Schaid, D. J., Chen, W. & Larson, N. B. From genome-wide associations to candidate causal variants by statistical fine-mapping. *Nat. Rev. Genet.* **19**, 491–504 (2018).

19. Sharma, A. & Palaniappan, L. Improving diversity in medical research. *Nat. Rev. Dis. Primers* **7**, 74 (2021).

20. Middleton, R. M. *et al.* Validating the portal population of the United Kingdom Multiple Sclerosis Register. *Mult. Scler. Relat. Disord.* **24**, 3–10 (2018).

21. Ford, D. V. *et al.* The feasibility of collecting information from people with Multiple Sclerosis for the UK MS Register via a web portal: characterising a cohort of people with MS. *BMC Med. Inform. Decis. Mak.* **12**, 73 (2012).

22. Huang, Q. Q. *et al.* Transferability of genetic loci and polygenic scores for cardiometabolic traits in British Pakistani and Bangladeshi individuals. *Nat. Commun.* **13**, 4664 (2022).

23. Finer, S. *et al.* Cohort Profile: East London Genes & Health (ELGH), a community-based population genomics and health study in British Bangladeshi and British Pakistani people. *Int. J. Epidemiol.* **49**, 20–21i (2020).

24. Bycroft, C. *et al.* The UK Biobank resource with deep phenotyping and genomic data. *Nature* **562**, 203–209 (2018).

25. Privé, F. Using the UK Biobank as a global reference of worldwide populations: application to measuring ancestry diversity from GWAS summary statistics. *Bioinformatics* **38**, 3477–3480 (2022).

For peer review only

26. Jacobs, B. M. *et al.* Modifiable Risk Factors for Multiple Sclerosis are Consistent Across Diverse Ethnic Backgrounds: A Nested Case-Control Study in a UK Population-Based Cohort. (2022) doi:10.2139/ssrn.4237729.

For peer review only

Full name	Primary affiliation
Alastair J Noyce	Preventive Neurology Unit, Wolfson Institute of Population Health, Queen Mary University of London
Angie Dunne	Leeds Centre for Neurosciences, Leeds teaching Hospitals NHS Trust
Antonio Scalfari	Centre of Neuroscience, Department of Medicine, Imperial College London
Benjamin M Jacobs	Preventive Neurology Unit, Wolfson Institute of Population Health, Queen Mary University of London
Bruno Gran	Department of Neurology, Nottingham University Hospitals NHS Trust; Mental Health and Clinical Neuroscience Academic Unit, University of Nottingham School of Medicine
Charles A Mein	Barts and the London Genome Centre, Queen Mary University of London
Charlotte Sellers	Preventive Neurology Unit, Wolfson Institute of Population Health, Queen Mary University of London
Cord Spilker	Bradford Teaching Hospital Foundation Trust
David Rog	Manchester Centre for Clinical Neurosciences, Northern Care Alliance NHS Trust
Eli Silber	Kings College Hospital and Lewisham and Greenwich NHS Trusts
Elisa Visentin	Research and Innovation, Queen's Hospital, BHRUT
Elizabeth Lindsey Bezzina	Kings College Hospital and Lewisham and Greenwich NHS Trusts
Emeka Uzochukwu	Division of Psychological Medicine and Clinical Neurosciences, Cardiff University
Emma Tallantyre	1. Division of Psychological Medicine and Clinical Neurosciences, Cardiff University<br>2. Department of Clinical Neurology, University Hospital of Wales, Cardiff.
Eva Wozniak	Barts and the London Genome Centre, Queen Mary University of London
Eve Sacre	Leeds Centre for Neurosciences, Leeds teaching Hospitals NHS Trust
Gavin Giovannoni	Preventive Neurology Unit, Wolfson Institute of Population Health, Queen Mary University of London
Helen L. Ford	Leeds Centre for Neurosciences, Leeds Teaching Hospitals NHS Trust
Huw Morris	Department of Clinical and Movement Neuroscience, UCL Queen Square Institute of Neurology, London UK
Jade Harris	Northern Care Alliance NHS Trust
Joshua Breedon	Preventive Neurology Unit, Wolfson Institute of Population Health, Queen Mary University of London
Judith Brooke	Northern Care Alliance NHS Trust
Karim L. Kreft	Department of Neurology, University Hospital of Wales, Cardiff
Katila George	Preventive Neurology Unit, Wolfson Institute of Population Health, Queen Mary University of London
Luisa Schalk	Preventive Neurology Unit, Wolfson Institute of Population Health, Queen Mary University of London
Martin O'Malley	Leeds Centre for Neurosciences, Leeds teaching Hospitals NHS Trust
Michelle Peter	NHS North Thames Genomic Laboratory Hub, Great Ormond Street Hospital for Children NHS Foundation Trust, London, UK
Miriam Mattoscio	Department of neuroscience, Queen's Hospital, BHRUT NHS Trust
Neisha Rhule	Queen Elizabeth Hospital (Lewisham and Greenwich NHS Trust)
Nimisha Vinod	Northern Care Alliance NHS Trust
Outi Quinn	Bradford Teaching Hospital Foundation Trust
Ramya Shamji	Research and Innovation, Queen's Hospital, BHRUT
Rashmi Kaimal	Preventive Neurology Unit, Wolfson Institute of Population Health, Queen Mary University of London
Rod Middleton	Population Data Science, Swansea University Medical School, Swansea
Roxanne Murray	Preventive Neurology Unit, Wolfson Institute of Population Health, Queen Mary University of London
Ruth Bellfield	Bradford Teaching Hospital Foundation Trust
Ruth Dobson	Preventive Neurology Unit, Wolfson Institute of Population Health, Queen Mary University of London
Sadid Hoque	Preventive Neurology Unit, Wolfson Institute of Population Health, Queen Mary University of London
Stephanie Mitchell	Northern Care Alliance NHS Trust
Stephen Sawcer	University of Cambridge, Department of Clinical Neuroscience, Addenbrookes Hospital, Hills Road, Cambridge, CB22 3TD
Tarunya Arun	University Hospitals of Coventry and Warwickshire
Tatiana Pogreban	Research and Innovation, Queen's Hospital, BHRUT
Terri-Louise Brown	Preventive Neurology Unit, Wolfson Institute of Population Health, Queen Mary University of London
Thamanna Begum	Preventive Neurology Unit, Wolfson Institute of Population Health, Queen Mary University of London
Veronica Antoine	Northern Care Alliance NHS Trust

For peer review only - http://bmjopen.bmj.com/site/about/guidelines.xhtml
